# Diffeomorphic registration using geodesic shooting and Gauss–Newton optimisation

**DOI:** 10.1016/j.neuroimage.2010.12.049

**Published:** 2011-04-01

**Authors:** John Ashburner, Karl J. Friston

**Affiliations:** Wellcome Trust Centre for Neuroimaging, UCL Institute of Neurology, London, UK

**Keywords:** Diffeomorphisms, Geodesic shooting, Shape modelling, Nonlinear registration, Gauss–Newton optimisation

## Abstract

This paper presents a nonlinear image registration algorithm based on the setting of *Large Deformation Diffeomorphic Metric Mapping* (LDDMM), but with a more efficient optimisation scheme — both in terms of memory required and the number of iterations required to reach convergence. Rather than perform a variational optimisation on a series of velocity fields, the algorithm is formulated to use a geodesic shooting procedure, so that only an initial velocity is estimated. A Gauss–Newton optimisation strategy is used to achieve faster convergence. The algorithm was evaluated using freely available manually labelled datasets, and found to compare favourably with other inter-subject registration algorithms evaluated using the same data.

## Introduction

This paper is about nonlinear image registration, which primarily aims to align images of different subjects, although it may also be of use for aligning longitudinal data of the same subject in situations where shape changes may have occurred. Inter-subject registration enables findings from functional imaging studies of different subjects to be brought within a common anatomical space, via a procedure known as “spatial normalisation”. In addition to this role, accurate alignment across subjects has many other applications, particularly in areas of translational science. Accurate registration allows information derived from some subjects (possibly from data that can only be collected post-mortem) to be generalised to the anatomy of other individuals.

Unfortunately, it is commonplace to find neuroimagers still using relatively old and inaccurate inter-subject registration techniques (Klein et al., 2009), which preclude accurate localisation of findings from multiple subjects. This may be because of a commonly held belief that brain anatomy is not predictive of brain function. There is increasing evidence emerging that shows this argument to be incorrect, and that by aligning anatomical features, such as cortical folds, we are able to also align functionality homologous areas. Relatively recent advances show that information from anatomical scans (such as T1-weighted MRI) do allow the underlying cyto-architecture to be predicted from folding patterns of the cortex (Fischl et al., 2008; Yeo et al., 2007). These studies were carried out by aligning cortical surfaces, and not by volumetric registration procedures. Evaluations based on manually traced structures show that nonlinear volumetric registration algorithms can be much more accurate than simple affine registration (Klein et al., 2009), although it still remains to be seen how well the most advanced volumetric registration methods can align cyto-architectonic borders. Klein et al. (2010) also showed that volumetric registration gave similar accuracy to cortical alignment approaches, although a more recent paper (Ghosh et al., 2010) showed higher accuracy for surface-based methods in some situations. The evaluations in the current paper will use some of the same dataset used by Klein et al. (2009), and are based on an assumption that manually drawn labels are accurate enough to be used as “ground truth”. Any gains in accuracy should be of benefit in terms of achieving greater overlap of functionally specialised brain regions across subjects. In addition to improved regional specificity to whatever measure is of interest, more accurate alignment should also provide increased sensitivity, with less need to spatially blur images in order to superimpose features.

Image registration models also play a useful role in geometric morphometrics, as registration essentially involves learning a model of the relative shapes of the organs or organisms under study. Shape, or form, may be encoded in numerous ways, some of which are more parsimonious than others. Under the assumption that measurements such as length, area and volume should all be positive, diffeomorphic registration approaches are able to encode relative shapes using the powerful *initial momentum* formulation (Wang et al., 2007; Younes, 2007). The decreasing cost of gene sequencing, along with a trend to assemble large datasets of scans, is likely to lead to renewed interest in modelling inter-subject variability. As outlined in Ashburner and Klöppel (2010), much of the inter-subject variance among brain images is dealt with by shape modelling (computational anatomy).

Any conclusions drawn from a study depend on how the data are modelled. In the case of computational anatomy studies, the accuracy of inter-subject registration plays a significant role in terms of the actual findings obtained, as well as on the interpretability of those findings. It is therefore worth ensuring that an accurate and coherent model of the data is used, before attempting to draw a conclusion from the fitted model. From a theoretical perspective, the state-of-the-art in terms of formulating volumetric image registration, in a mathematically coherent way, is probably the *Large Deformation Diffeomorphic Metric Mapping* (LDDMM) of Beg et al. (2005).

Most image registration methods are based on a small-deformation approximation, which attempts to represent relative shapes in terms of displacement fields. Such models assume that displacements may be added and subtracted in a linear way, rather than by correctly composing deformations. Assumptions of linearity result in a number of problems (one-to-one mappings break down, lack of inverse consistency, etc), which are generally either ignored, or fixed using ad hoc procedures. The LDDMM framework resolves these limitations, at source, by using a more coherent formulation of the registration model. Instead of incorrectly assuming linearity, the formulation incorporates established techniques from the fields of differential geometry and mechanics.

Another commonly used framework is the one known as “viscous-fluid modelling” (Christensen et al., 1996), which does not have a clearly defined objective function, thus precluding a probabilistic interpretation of the model. This is likely to limit its long term applicability.

This paper builds on LDDMM, but includes some additional components that are intended to enable more efficient registration, both in terms of the number of iterations needed to achieve convergence and also the amount of memory required for encoding the deformations. Although over the longer term, processing speed will become much less important than accuracy, it is still worth trying to achieve equally accurate results as efficiently as possible.

## Methods

In the current work, image registration is treated as an optimisation problem, which involves minimising an objective function consisting of the sum of two terms.

The first term is a measure of how much the template is distorted in order to match the individual's image. Because deformations do not add and subtract linearly, it is not optimal to measure the magnitude of a deformation based on some linear measure computed from a single displacement field. Such small-deformation approximation approaches are commonly used, but they do not give consistent measures of deformation magnitude between forward and inverse deformations. The magnitude of a deformation is better computed as a geodesic distance, using ∫ _*t* = 0_^1^||**Lv**_*t*_||*dt*, where **L** is a linear operator, which operates on a time-dependent velocity that mediates the deformation over unit time. In practice, the registration is regularised by penalising the “energy” in the deformation ($\frac{1}{2}\int_{t=0}^{1}||Lv_{t}|{|}^{2}dt$), where **L** determines the nature of the energy (based on beliefs about what sorts of deformations are more probable *a priori*). Occasionally, the literature refers to velocities where each point in the time varying velocity field (**v**_*t*_) is associated with the same point in the underlying image. This is not the case here, as **v**_*t*_ is the Eulerian speed vector field, defined over the ambient space through which the deforming image passes.

The second term is a measure of how closely the images appear to be aligned, and is typically one of the usual cost functions used for image registration, such as the mean squared difference between a subject's image (*f*) and a deformed version of the template (*μ*(*φ*_1_^− 1^)). Here, *φ* is a diffeomorphic mapping (diffeomorphism) encoding the deformation. With this image matching term, the algorithm minimises the following:

(1) $$E=\frac{1}{2}\int_{t=0}^{1}||Lv_{t}|{|}^{2}dt+\frac{1}{2\sigma^{2}}||f-\mu \left(\varphi_{1}^{-1}\right)|{|}^{2}\text{,}\;\text{where}\;\varphi_{0}=\text{Id}\text{,}\;\frac{d\varphi }{dt}=v_{t}\left(\varphi_{t}\right)$$

Computing a diffeomorphic deformation is treated as modelling a dynamical system, which evolves over unit time. Subscripts on **v** and *φ* indicate velocity fields and diffeomorphisms at different time points. The easiest way to conceptualise the evolution is in terms of an Euler integration, in which case the diffeomorphism (*φ*_1_) and its inverse (*ϑ*_1_) are computed from the compositions of series of small-deformations. From this perspective, a series of *N* velocity fields are used to represent the time varying velocity field. For *N* uniformly spaced time steps (0, *t*_1_, *t*_2_,..., *t*_*N* − 2_, *t*_*N* − 1_), computing the diffeomorphisms may be achieved by:

(2) $$\varphi_{1}=\left(\text{Id}+\frac{1}{N}v_{t_{N-1}}\right)\circ \left(\text{Id}+\frac{1}{N}v_{t_{N-2}}\right)\circ ...\circ \left(\text{Id}+\frac{1}{N}v_{t_{1}}\right)\circ \left(\text{Id}+\frac{1}{N}v_{0}\right)$$

(3) $$\vartheta_{1}=\left(\text{Id}-\frac{1}{N}v_{0}\right)\circ \left(\text{Id}-\frac{1}{N}v_{t_{1}}\right)\circ ...\circ \left(\text{Id}-\frac{1}{N}v_{t_{N-2}}\right)\circ \left(\text{Id}-\frac{1}{N}v_{t_{N-1}}\right)$$

Providing all the small-deformations are sufficiently small to be one-to-one (and satisfy certain smoothness criteria), their compositions should also result in one-to-one mappings (Christensen et al., 1995). More sophisticated integration methods (than Euler) yield more accurate results using fewer time steps, but are not explored here. It should also be pointed out that care should be taken with the compositions, particularly when interpolating deformation fields close to boundaries. In most situations, it is more efficient to use $\varphi +\frac{1}{N}v_{t}\circ \varphi$ instead of $\left(\text{Id}+\frac{1}{N}v_{t}\right)\circ \varphi$.

Beg et al. (2005) describe registration in terms of a variational optimisation of this sequence of velocity fields, using a gradient descent scheme. This approach has two main disadvantages.

1. The entire sequence of velocity fields needs to be retained, either in memory or on disk, which can make the approach quite demanding in terms of memory requirements.
2. Gradient descent optimisation is slow, and requires many iterations to reach satisfactory convergence.

Instead of using a variational scheme to estimate a series of velocity fields, the aim of the optimisation in the current work is to determine only an initial velocity field (**v**_0_). Forward and backward deformations (*φ* and *ϑ*) may then be computed from the initial velocity, using a *geodesic shooting* scheme. The use of GS negates the need to store the entire series of velocity fields, thus reducing memory and disk space requirements. The reason this works is that the principle of stationary action uniquely determines the trajectory of the deformation, given the initial velocity. Furthermore, because (kinetic) energy is conserved, we only need to evaluate the energy for this initial velocity. A related scheme has already been devised by Marsland and McLachlan (2007), who parameterised two-dimensional deformations using 21 control points. Registration then involved estimating the 42 parameters that encode the initial momenta of these points. As pointed out by Marsland, his framework is too computationally expensive to use many control points and therefore not practical for the six million or so parameters that we use to represent relative shapes. A similar framework for optimising initial momentum was also presented in Cotter and Holm (2006), but involved a particle mesh method that overcomes many of the computational problems of using control points. The work presented here shares a great deal with that in Cotter and Holm (2006) (neither requires the entire sequence of velocity fields to be stored), and is essentially a Gauss–Newton implementation of that approach.

In the current work, registration is treated as a nonlinear optimisation problem, where the aim is to determine the optimal values for the coefficients parameterising a discretised version of the initial velocity field. Because it is nonlinear and has no closed-form solution, it requires an iterative approach to solve. We use a Gauss–Newton optimisation scheme, which uses approximations to both first and second derivatives and usually achieves convergence in fewer iterations than an approach using only first derivatives.

The next section describes geodesic shooting, and this is followed by a section describing the optimisation scheme.

### Geodesic shooting

Beg's algorithm may be conceptualised within the framework of the *principle of stationary action*, which is a variational principle that may be used for obtaining equations of motion. Within this framework, **L**^†^**L** may be considered as a model of the “inertia” of the system, such that the “kinetic energy” of the evolving system is given by $\frac{1}{2}\langle v_{t},L^{†}Lv_{t}\rangle$. Similarly, there is a concept of momentum, given by **u**_*t*_ = **L**^†^**Lv**_*t*_. Velocity may be derived from momentum by smoothing with **K**, which is the inverse of the **L**^†^**L** operator. In other words, **KL**^†^**Lv** = **v** and **L**^†^**LKu** = **u**. Given an initial and final configuration (ie an identity transform and the final deformation respectively) at each iteration, Beg's algorithm determines the series of intermediate configurations that have the least kinetic energy. In practice it is a little more complicated than that, as the estimation of the final configuration is not really separated from the estimation of the intermediate configurations. The solution obtained by LDDMM satisfies the condition that the derivatives of the objective function with respect to changes in the velocity are zero. These derivatives were derived in Beg et al. (2005), and a simpler derivation was also given in the appendix of Ashburner (2007). This solution obeys the following *Euler–Lagrange equation* (Eq. (9) of Beg et al. (2005)), where the **D** operator refers to computing the Jacobian tensor:

(4) $$v_{t}+K\left(\frac{1}{\sigma^{2}}|D(\varphi_{1}\circ \varphi_{t}^{-1})|(\nabla (\mu \circ \varphi_{t}^{-1}))(f\circ \varphi_{1}\circ \varphi_{t}^{-1}-\mu \circ \varphi_{t}^{-1})\right)=0$$

The foregoing equation shows that, at the solution, the velocity at each time point may be derived from the initial velocity. The gradients of the warped template ∇ (*μ* ∘ *φ*_*t*_^− 1^) may also be computed by warping the gradients of the template and multiplying by the transpose of the Jacobian tensor at each point (**D***φ*_*t*_^− 1^)^*T*^((∇ *μ*) ∘ *φ*_*t*_^− 1^). Also, the Jacobian determinants of the composed transformations |**D**(*φ*_1_ ∘ *φ*_*t*_^− 1^)| may by computed by (|**D***φ*_1_| ∘ *φ*_*t*_^− 1^)|**D***φ*_*t*_^− 1^|. This leads to the following re-arrangement of Eq. (4):

(5) $$v_{t}=K\left(|D\varphi_{t}^{-1}|{\left(D\varphi_{t}^{-1}\right)}^{T}\left(\left(\frac{1}{\sigma^{2}}|(D\varphi_{1}|(\nabla \mu )(\mu -f\circ \varphi_{1})\right)\circ \varphi_{t}^{-1}\right)\right)$$

At time zero, *φ*_0_ is the identity transform, so the initial momentum is:

(6) $$u_{0}=L^{†}Lv_{0}=\frac{1}{\sigma^{2}}|D\varphi_{1}|(\nabla \mu )(\mu -f\circ \varphi_{1})$$

Combining Eqs. (5) and (6) shows that the velocity at any time is given by the initial velocity or momentum:

(7) $$v_{t}=K\left(\left|D\varphi_{t}^{-1}|{\left(D\varphi_{t}^{-1}\right)}^{T}(u_{0}\circ \varphi_{t}^{-1}\right)\right)$$

This conservation of momentum is well known and leads to an alternative approach, which is to formulate each iteration of the registration as an initial value problem. Here, the intermediate configurations, and therefore the final deformation, are all computed from the initial conditions. These initial conditions are the spatial configuration (an identity transform) and the initial velocity or momentum. This procedure is known as *geodesic shooting* (GS), and may be viewed as an integration based on Hamilton's equations. More complete explanations of the mathematics underlying the GS approach are to be found in the literature (Miller et al., 2006; Cotter and Holm, 2006; Marsland and McLachlan, 2007; Younes, 2007; Younes et al., 2008, 2009) or in various textbooks (Younes, 2010; Holm et al., 2009; Grenander and Miller, 2007). This section will simply outline how a deformation and its inverse may be computed from an initial velocity field, by Euler integration.

Geodesic shooting requires the initial momentum (**u**_0_), which is derived from the initial velocity by applying **L**^†^**L**.

(8) $$u_{0}=L^{†}Lv_{0}$$

The inverse (backward) deformations are initialised to the identity and, if required, their Jacobian tensor fields are set to an identity matrix at each point. Here, the **D** operator is used to denote computing the Jacobian tensor at each point in the image. In this case, the Jacobian tensors from an identity transform are all identity matrices.

(9) $$\vartheta_{0}=\text{Id}\text{,}\;J_{0}^{\vartheta }=D\vartheta_{0}$$

If required, the forward deformation is also initialised to an identity transform, and possibly also its Jacobian tensor field.

(10) $$\varphi_{0}=\text{Id}\text{,}\;J_{0}^{\varphi }=D\varphi_{0}$$

Then the following (Eqs. (11) to (16)) are executed for each of *N* time steps. For the *n*th time step, the backward deformation is incremented by composing it with a small-deformation.

(11) $$\vartheta_{t_{n}}=\vartheta_{t_{n-1}}\circ \left(\text{Id}-\frac{1}{N}v_{t_{n-1}}\right)$$

This procedure requires the Jacobians of this deformation. These may be constructed from the sequential composition of the Jacobians of the small-deformations, but may also be derived by computing the gradients of *ϑ*_*t*_*n* − 1__. The procedure involves matrix multiplications with the 3 × 3 Jacobian tensors at each point.

(12) $$J_{t_{n}}^{\vartheta }={\left(J_{t_{n-1}}^{\vartheta }\circ \left(\text{Id}-\frac{1}{N}v_{t_{n-1}}\right)\right)}^{T}\left(D\left(\text{Id}-\frac{1}{N}v_{t_{n-1}}\right)\right)$$

A forward deformation and its Jacobian tensor field may be required, but it is not strictly necessary for the integration.

(13) $$\varphi_{t_{n}}=\left(\text{Id}+\frac{1}{N}v_{t_{n-1}}\right)\circ \varphi_{t_{n-1}}$$

(14) $$J_{t_{n}}^{\varphi }={\left(\left(D\left(\text{Id}+\frac{1}{N}v_{t_{n-1}}\right)\right)\circ \varphi_{t_{n-1}}\right)}^{T}J_{t_{n-1}}^{\varphi }$$

The velocity field is updated, by first generating a view of the momentum, which accounts for the current deformation.

(15) $$u_{t_{n}}=|J_{t_{n}}^{\vartheta }|{\left(J_{t_{n}}^{\vartheta }\right)}^{T}\left(u_{0}\circ \vartheta_{t_{n}}\right)$$

Velocity is then obtained from the momentum by applying the K operator. Fourier transform methods may be used to effect this convolution, but other approaches, such as the multi-grid methods used in the current paper, are also possible.

(16) $$v_{t_{n}}=Ku_{t_{n}}$$

#### An alternative integration scheme

The registration algorithms described in this paper use an alternative integration scheme, which is now presented. Rather than transforming the initial momentum using *ϑ*_*t*_ with a pullback scheme, it uses *φ*_*t*_ with a push-forward. It is therefore more suited to the direct computation of *φ*_1_ from $v_{0}$.

The procedure begins by computing the initial momentum from its velocity (Eq. (8)), and setting the forward deformation and its Jacobian tensor field to identity transforms (Eq. (10)). Then the following (Eqs. (17) to (20)) are computed for each of the *N* time steps.

Update the forward deformation using Eq. (13).

(17) $$\varphi_{t_{n}}=\left(\text{Id}+\frac{1}{N}v_{t_{n-1}}\right)\circ \varphi_{t_{n-1}}$$

In this integration strategy, the inverses of the Jacobian matrices at each point will be used. If relatively few time steps are used, the possibility of small-deformations containing Jacobians with zero or negative determinants becomes more likely. To increase stability, the computation of the Jacobian tensor field is therefore modified slightly, replacing the small-deformation approximation of the Jacobians by the matrix exponentials (eg, see Moler and Van Loan (2003)) of the gradients at each point of the velocity field. The use of matrix exponentials is to ensure that the Jacobians are invertable (by preventing their determinants from approaching zero), even though the small-deformation itself may not have positive Jacobian determinants.

(18) $$J_{t_{n}}^{\varphi }={\left(\text{Exp}\left(\frac{1}{N}Dv_{t_{n-1}}\right)\circ \varphi_{t_{n-1}}\right)}^{T}J_{t_{n-1}}^{\varphi }$$

Obtaining the new view of the momentum involves a push-forward scheme. This will be denoted by $\varphi^{⁎}u$, and involves adding each of the voxels in u into the appropriate positions of the warped version. The end result is similar to $|J^{\varphi^{-1}}|\left(u\circ \varphi^{-1}\right)$, but contains some aliasing effects.

(19) $$u_{t_{n}}=\varphi_{t_{n}}^{⁎}\left({\left({\left(J_{t_{n}}^{\varphi }\right)}^{-1}\right)}^{T}u_{0}\right)$$

The final procedure within each time step is to update the velocity (Eq. (16)).

(20) $$v_{t_{n}}=Ku_{t_{n}}$$

### Optimisation

In this work, registration is viewed as an optimisation procedure, where the objective is to estimate the initial velocity field, parameterising the diffeomorphism that best aligns the images. An optimisation scheme based on using approximations to both first and second derivatives is presented. It will be described for a matching term based on the sum of squares difference, but other objective functions may also be used.

Conservation of “kinetic energy” allows the registration objective function to be formulated as:

(21) E=E1+E2=12||Lv0||2+12σ2∫x∈Ωf(x)−μ(φ1−1(x))2dx

This objective function can be re-written as the difference between the template and warped image, by including a change of variables to account for expansion and contraction.

(22) E=12||Lv0||2+12σ2∫x∈Ω|J1φ(x)|f(φ1(x))−μ(x)2dx

**Fig. 1:**
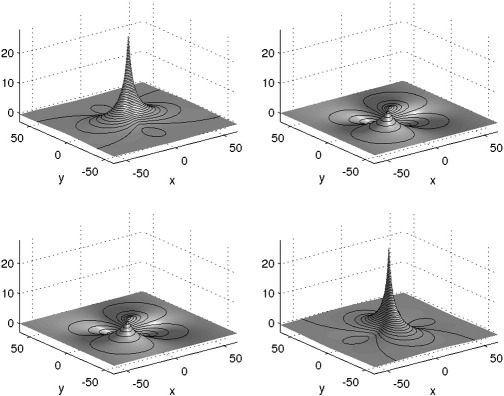
The inverse of the elasticity operator, which is used for computing velocity from momentum (**v**_*t*_ = **Ku**_*t*_). This is the Green's function (fundamental solution for a linear partial differential operator). Note that this figure shows a 2D version of the operator. Obtaining the *x* (horizontal) component of the velocity involves convolving the *x* component of the momentum with the function shown at the top left, and adding the *y* (vertical) component of the momentum, convolved with the function shown at the top right. Similarly, obtaining the velocity's *y* component is by convolving the momentum's *x* component with the lower-left function, and adding this to the momentum's *y* component convolved with the lower-right function.

For each iteration of LDDMM, all the relevant deformations (*ϑ*_*t*_*n*__ and *φ*_1_) are computed from the current estimates of the velocity fields ($v_{t_{n}}^{\mathrm{iter}}$), and then the velocity fields are updated by a descent step (scaled by *ε*) along the, so called, Hilbert gradient. Briefly, the Hilbert gradient may be considered as the derivatives of the objective function with respect to variations in the velocity, if this velocity were parameterised by a linear combination of Green's functions similar to those shown in Fig. 1. Without including the K operator in the update equations (to give the Hilbert gradient), the gradient descent would be much less stable. In the following update equation, the multiplications by ${\left(J_{t_{n}}^{\vartheta }\right)}^{T}$ account for the changes to the template gradients as it is warped over time (see later). Similarly, Jacobian determinants are included because of the change of variables needed to account for expansion or contraction of the individual image. The following gradient descent step is simply a re-expression of Eqs. (10) and (12) of Beg et al. (2005).

(23) $$v_{t_{n}}^{iter+1}=v_{t_{n}}^{\mathrm{iter}}-\varepsilon K\left(L^{†}Lv_{t_{n}}^{\mathrm{iter}}+|J_{t_{n}}^{\vartheta }|{\left(J_{t_{n}}^{\vartheta }\right)}^{T}\left(\left(\frac{\left|J_{1}^{\varphi }\right|}{\sigma^{2}}(f\circ \varphi_{1}-\mu )\nabla \mu \right)\circ \vartheta_{t_{n}}\right)\right)$$

This procedure, which involves alternating between updating all the deformations, and updating all the velocities, is repeated until convergence or until some limit on the number of iterations is reached.

Differentiating *φ*_1_ with respect to variations in $v_{0}$ is not straightforward, when it is computed via GS. This leads to difficulties in computing the exact derivatives needed for Gauss–Newton optimisation. Therefore, an alternative strategy is adopted. First of all though, the principles of how the initial velocity could be optimised using gradient descent will be illustrated. Simplifying Eq. (23) for the special case of the initial velocity gives the following gradient descent step.

(24) $$v_{0}^{iter+1}=v_{0}^{\mathrm{iter}}-\varepsilon K\left(L^{†}Lv_{0}^{\mathrm{iter}}+\left(\frac{\left|J_{1}^{\varphi }\right|}{\sigma^{2}}(f\circ \varphi_{1}-\mu )\nabla \mu \right)\right)$$

In essence, the LDDMM algorithm (Beg et al., 2005) updates $v_{0}$ using Eq. (24), and would normally proceed to update the remaining velocity fields using Eq. (23). However, rather than updating the remaining fields by gradient descent, they could instead be updated by shooting from $v_{0}$. This is a similar procedure to that employed in Cotter and Holm (2006) and Marsland and McLachlan (2007). Providing the gradient descent step on the initial velocity brings it closer to its optimal solution, the updates of the remaining velocity fields should also be brought closer to their optima.

The Gauss–Newton approach is now described, which uses both first and second derivatives. To make the problem tractable, at each iteration the update can be conceptualised as estimating a small displacement field (s) that would improve the objective function. The estimated displacement is treated as an increment to the initial velocity, which is then used to update the deformation via geodesic shooting. Deriving the first and second derivatives necessary for each iteration of this approach involves differentiating the following (around s=0), with respect to variations in s (while holding $v_{0}$ and $\varphi_{1}$ fixed):

(25) $$E={\frac{1}{2}||L(v_{0}+s)|{|}^{2}|}_{s=0}+{\frac{1}{2\sigma^{2}}\int_{x\in \Omega }|J_{1}^{\varphi }(x)|{\left(f(\varphi_{1}(x))-\mu (x-s(x))\right)}^{2}dx|}_{s=0}$$

Because it is often easier to discretise the problem prior to optimising, the descriptions in the remainder of this section will use a discrete formulation. The initial velocities are now represented as a linear combination of trilinear interpolation basis functions. The value of each point (x) in the continuous vector field ($v_{0}(x)$) is encoded by $\sum_{i=1}^{I}\;w_{i}b_{i}(x)$, where $b_{i}(x)$ is the *i*th basis function. Similarly, s(x) in Eq. (25) is parameterised the same way. The registration involves estimating the vector of *I* coefficients w. Within the discrete setting, $\frac{1}{2}||Lv_{0}|{|}^{2}$ may be computed by $\frac{1}{2}w^{T}Aw$, where A is a very large sparse matrix encoding the operator $L^{†}L$. See, for example, Modersitzki (2009) for further details about how such operators may be formulated as matrices. Within this discrete setting, the gradient descent update in Eq. (24) may be expressed as:

(26) $$w^{iter+1}=w^{\mathrm{iter}}-\varepsilon A^{-1}\left(Aw^{\mathrm{iter}}+g^{\mathrm{iter}}\right)$$

For the 3D case, the vector of first derivatives may be written in terms of its three components as:

(27) $$g=\left[\begin{array}{c} g^{\left(1\right)}\\ g^{\left(2\right)}\\ g^{\left(3\right)} \end{array}\right]$$

The velocity is parameterised using trilinear interpolation basis functions, so using ∇ _*l*_ to indicate the gradient along the *l*th dimension, the components of the derivatives are computed by:

(28) $$g_{i}^{\left(l\right)}=\frac{dE_{2}}{dw_{i}}=\frac{1}{\sigma^{2}}\left|J_{1}^{\varphi }\left(x_{i}\right)\right|\left(f\left(\varphi_{1}\left(x_{i}\right)-\mu \left(x_{i}\right)\right)\right)\left(\left(\nabla_{l}\mu \right)\circ x_{i}\right)$$

Convergence of gradient descent algorithms is often much slower than that of algorithms that also use second derivatives. By including an approximation of the Hessian of $E_{2}$ within the optimisation, it is possible to make the update steps more effective. Including the Hessian (H) to obtain a Gauss-Newton optimisation involves a slight change to Eq. (26).

(29) $$w^{iter+1}=w^{\mathrm{iter}}-\gamma {\left(A+H^{\mathrm{iter}}\right)}^{-1}\left(Aw^{\mathrm{iter}}+g^{\mathrm{iter}}\right)$$

The foregoing equation is a slightly modified version of the pure Gauss–Newton update formula, as it includes a scaling parameter (*γ*), which may be used to prevent updates from overshooting. For a pure Gauss–Newton approach, *γ* would be set to 1, but there may be situations where its value should be decreased. For example, after an iteration in which the objective function gets worse, it can be a good idea to halve the value of *γ*. This situation can occur with the diffeomorphic registration procedure, but it also happens when optimising small-deformation registration models.

Instead of the true Hessian (of Eq. (25)), a positive semi-definite approximation is used, that ignores derivatives of the template that are higher than first order (see eg Modersitzki (2009)). Just as the first derivatives (g) may be computed by differentiating Eq. (25) around s=0, so the Hessian (H) may be computed in a similar way. Again, because the velocity field is modelled using trilinear interpolation, these second derivatives of $E_{2}$ (based on Eq. (25)) have the following form:

(30) $$H=\left[\begin{array}{ccc} \text{diag}\left(h^{\left(11\right)}\right) & \text{diag}\left(h^{\left(12\right)}\right) & \text{diag}\left(h^{\left(13\right)}\right)\\ \text{diag}\left(h^{\left(12\right)}\right) & \text{diag}\left(h^{\left(22\right)}\right) & \text{diag}\left(h^{\left(23\right)}\right)\\ \text{diag}\left(h^{\left(13\right)}\right) & \text{diag}\left(h^{\left(23\right)}\right) & \text{diag}\left(h^{\left(33\right)}\right) \end{array}\right]$$

where:

(31) $$h_{i}^{\left(lm\right)}=\frac{1}{\sigma^{2}}|J_{1}^{\varphi }(x_{i})|((\nabla_{l}\mu )\circ x_{i})((\nabla_{m}\mu )\circ x_{i})$$

The overall algorithm is summarised as follows.

• Set the initial velocity $v_{0}$ (parameterised by w) to zero, and *γ* to 1.
• Repeat the following until convergence or for a fixed number of iterations
  - Shoot from the initial velocity $v_{0}$ to obtain *φ*_1_.
  - Compute the objective function, and approximate gradient and Hessian (E, g and H), using the current *φ*_1_. These are in Eqs. (22), (27) and (30).
  - If E is worse than that from the previous iteration, decrease *γ*.
  - The coefficients, which parameterise $v_{0}$, are updated using Eq. (29).

The Gauss–Newton updates involve very large sparse matrices. Various numerical optimisation techniques may be used for computing ${\left[H+A\right]}^{-1}\left[g+Aw\right]$, many of which are outlined by Modersitzki (2009). A multi-grid approach was used for the work described in this paper, which was the same implementation as used in Ashburner (2007).

## Results and discussion

This paper is concerned with increasing the efficiency of LDDMM, and focuses on one aspect of image registration. The aim here is simply to demonstrate some of the desirable properties of the algorithm, and to assess the accuracy of the resulting image alignment. A two-dimensional toy example is provided next, which illustrates some of the properties of the resulting deformations. This is followed by an evaluation of the label propagation accuracy obtained when the algorithm is applied to real three-dimensional brain images. Then there is an illustration of the rate of convergence with real three-dimensional data, which is followed by the final section demonstrating some of the invariance properties of the GS formulation.

### Two-dimensional example

**Fig. 2:**
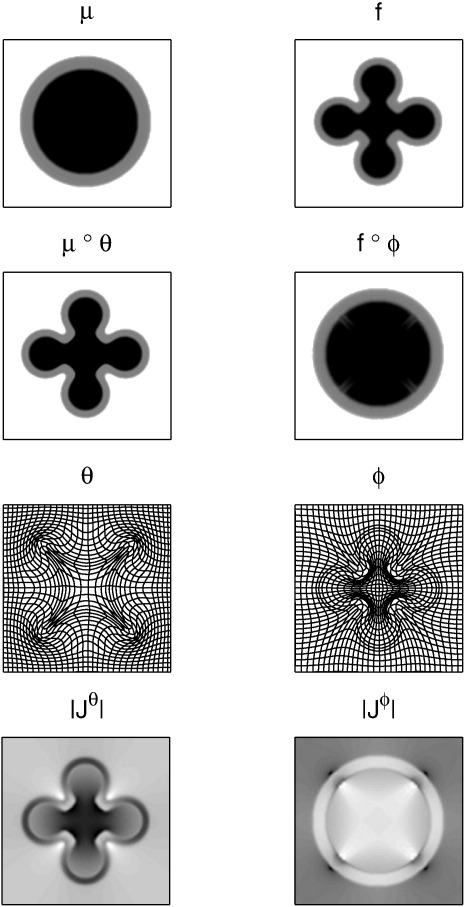
Results of diffeomorphic registation of two simulated images. Original images (top row), registered images (2nd row), diffeomorphic deformations (3rd row) and Jacobian determinants (bottom).

**Fig. 3:**
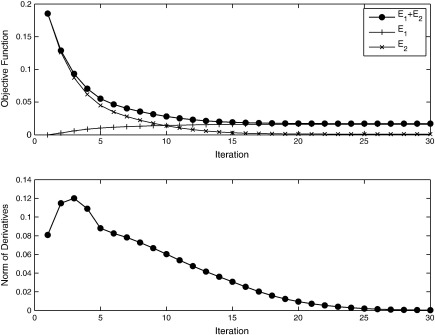
Convergence of the Gauss–Newton optimisation. The top panel shows how the objective function is reduced at each iteration, whereas the lower panel shows the norm of the derivatives of the objective function with respect to the model parameters. At the exact solution (either globally or locally optimal), this norm should be zero.

Two simulated two-dimensional images (128 × 128 pixels) were registered together to illustrate the underlying principles. An image containing two concentric circles was used as the template (*μ*), and the target (*f*) was an image of a more complex shape (shown in Fig. 2). The objective function was the sum of squares difference between the target and warped template images, and the operator ($L^{†}L$) encoded linear elasticity (as used by Christensen et al. (1996)). The boundary conditions were circulant, and the Euler integration used 20 time steps. To illustrate the effectiveness of the Gauss–Newton approach, Fig. 3 shows a plot of the objective function with each iteration. For this example, a reasonably accurate solution is achieved within about 20 to 30 iterations.

**Fig. 4:**
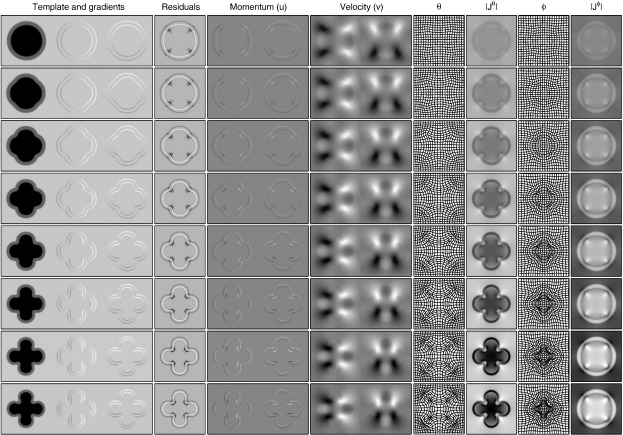
Illustration of the evolution equations for computing diffeomorphisms. The top row shows the system at time zero, which is followed in successive rows at later time points. Note that only eight time points were used for this integration, and that images are scaled so that intensities range between the overall minimum and maximum values within each column. Darker regions indicate larger values.

Fig. 4 illustrates the evolution equations that construct diffeomorphic deformations from an initial velocity or momentum field. The first column shows the template as it is deformed over time (*μ* ∘ *ϑ*_*t*_*n*__), and its horizontal and vertical spatial gradients (∇ (*μ* ∘ *ϑ*_*t*_*n*__), which may also be computed by ${\left(J_{t_{n}}^{\vartheta }\right)}^{T}((\nabla \mu )\circ \vartheta_{t_{n}})$). This is followed by a column of residual images, constructed from $\frac{1}{\sigma^{2}}|J_{t_{n}}^{\vartheta }|((|J_{1}^{\varphi }|(\mu -f\circ \varphi_{1}))\circ \vartheta_{t_{n}})$. Next is the momentum at different time points, which may be constructed by multiplying the warped residuals by the gradients of the warped template. Obtaining the velocity fields (v) from the momentum is by applying K (Eqs. (16) or (20)), which is essentially a convolution with the function shown in Fig. 1. These time varying velocity fields are shown in the next column. Updates to the backward and forward deformations may then be made by composing with small-deformations constructed using this velocity field (Eqs. (11) to (14)). These deformations, along with their Jacobian determinants are shown in the final four columns.

#### Comparison with some other parameterisations

**Fig. 5:**
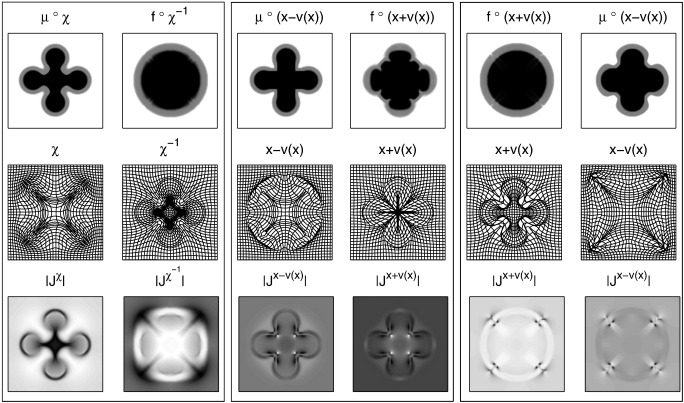
Various other deformation model results. Left panel: registration using a log-Euclidean model (Eq. (33)). Centre panel: small-deformation of the template to the individual (Eq. (34)). Right panel: small-deformation of the individual to the template (Eq. (35)). Note that the Jacobian determinant images are shown scaled between their minimum and maximum values and that darker regions indicate larger values.

The same 2D examples were also registered using some other approaches, with the aim of illustrating some of the limitations that are overcome using the diffeomorphic formulation. The first of these involved parameterising with a one-parameter subgroup, which allows diffeomorphic mappings to be constructed via a scaling and squaring procedure (Arsigny et al., 2006; Ashburner, 2007; Arsigny et al., 2009). It was intended to serve as a fast approximation to the full diffeomorphic framework described in this work. An inverse consistent formulation was used, which involved minimising the following

(32) $$E_{\mathrm{ops}}=\frac{1}{2}||Lv|{|}^{2}+\frac{1}{4\sigma^{2}}\int_{x\in \Omega }\;{\left(f(x)-\mu (\chi^{-1}(x))\right)}^{2}dx+\frac{1}{4\sigma^{2}}\int_{x\in \Omega }\;{\left(\mu (x)-f\left(\chi \left(x\right)\right)\right)}^{2}dx$$

where **χ** is computed by integrating χ̇ = **v(χ)** over unit time, after initially setting **χ** to an identity transform. The inverse (*χ*^− 1^) may be computed by simply reversing the sign of v. Eight squaring steps were used, which corresponds to an Euler integration with 256 time steps. The same linear elasticity metric was used as a regulariser and also the same value of *σ*^2^. The results of this registration are presented in the left-hand panel of Fig. 5, and show that the log-Euclidean approximation achieves a reasonably good overlap between the two images. The log-Euclidean approximation is unable to encode all possible diffeomorphic mappings (see page 456 of Kriegl and Michor (1997)), so the model had to introduce additional distortions to achieve this overlap. This is particularly visible in the Jacobians when they are compared to those in Fig. 2. It is readily apparent that the log-Euclidean approach does not localise volumetric differences as accurately as the shooting approach. This is likely to make the GS approach more suited to morphometric applications.

Two small-deformation models were also included (both using the same regularisation), the first of which involved warping the template to match the individual. The displacement field (v) was found that minimises

(33) $$E_{sd1}=\frac{1}{2}||Lv|{|}^{2}+\frac{1}{2\sigma^{2}}\int_{x\in \Omega }\;{\left(f(x)-\mu (x-v(x))\right)}^{2}dx$$

Registration results from this model are presented in the centre panel of Fig. 5 and show that this model was unable to achieve a good overlap between the images. When compared with the results in Fig. 2, it should be readily apparent that the inverse of a deformation cannot be achieved by negating a displacement field. This illustrates the fact that combined deformations cannot be computed accurately by simply adding or subtracting displacement fields, and therefore that the study of shapes cannot be optimally achieved using simple linear models. Another issue is that the resulting Jacobian determinants were not all positive, indicating that the one-to-one mapping has broken down and the deformations are not invertable. Negative Jacobian determinants also pose a problem for morphometric applications that involve working with logarithms of Jacobians. Also of note is the fact that the Jacobian determinants are not in alignment with the template image, which is another reason why this approach may be unsuited to morphometric applications.

The second small-deformation model involved warping the individual to the template, by minimising the following.

(34) $$E_{sd2}=\frac{1}{2}||Lv|{|}^{2}+\frac{1}{2\sigma^{2}}\int_{x\in \Omega }\;{\left(f(x+v(x))-\mu (x)\right)}^{2}dx$$

This formulation of a small-deformation model is less correct from a generative modelling perspective, as it does not allow an image to be treated as a sample from the probability density encoded by the model. However, it is an approach that is commonly used for spatially normalising multiple images to the same template. The results of this model are illustrated in the right-hand panel of Fig. 5, and again show that linear addition and subtraction of displacement fields is not appropriate. Also, some parts of the deformation fields had negative Jacobian determinants, which show the one-to-one mapping breaking down. The resulting deformation fields from Eq. (35) are more suited to some morphometric applications than those of (34).

**Fig. 6:**
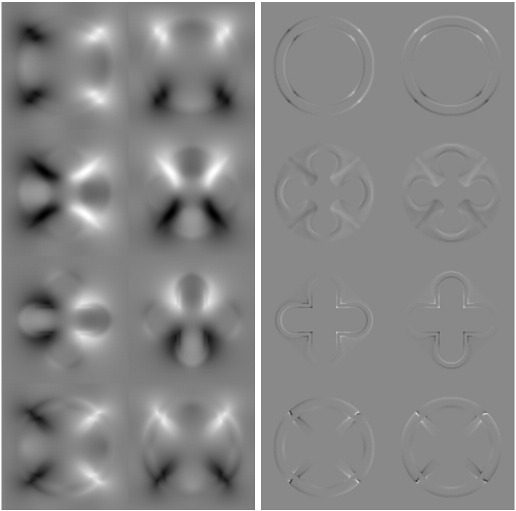
The velocity (left panel) and “momentum” (right panel) fields of the four models. The left column shows the horizontal component, whereas the right column shows the vertical component. The top row shows the initial velocities and momenta obtained using the shooting method. Velocities and momenta from the log-Euclidean method (Eq. (33)) are shown in the second row. Those from the small-deformation methods are shown in the third (Eq. (34)) and fourth (Eq. (35)) rows.

Fig. 6 shows the parameters of the various models, illustrating the fact that the shooting method aligns shape information with the template image. For morphometric applications, where images of multiple subjects are aligned to a common template, this alignment of information should lead to a more parsimonious representation when using approaches such as principal component analysis.

### Comparison with human expert segmentation

Evaluation was performed using similar procedures to those of Klein et al. (2009), and involved two datasets that are publicly available. Although these datasets do not provide absolute ground truth, they do allow automated methods to be compared against human experts. All the subjects’ scans have manually defined labels associated with them, which enables a comparison between manual and automatic structure labelling. For each of the datasets, the procedure involved aligning all the MR scans together (without using knowledge of the structure labels), and assessing how close the alignment is by warping each subject's structure labels into alignment with each other subject's labels. Overlap measures are most meaningful when compared with those achieved by other approaches, so the reader is referred to Klein et al. (2009) for reports of the “target overlap” measures from 15 other inter-subject registration algorithms. The measure is defined by the volume over which the deformed source labels match the target labels, divided by the total volume of the target labels.

In the Klein paper, registration was done in a pairwise manner. In this evaluation, registration is between each individual in a dataset and the common average shaped template for that dataset. Rather than aligning the images themselves, the registration aligned tissue class data, and assumed that the tissue images of each subject are drawn from a multinomial distribution, whose mean is represented by a deformed version of the template (Ashburner and Friston, 2009). For *M* tissue classes, over *I* voxels, the objective function to minimise for one image is:

(35) E=12||Lv0||2−∑i=1I|J1φ(xi)|∑m=1Mfm(φ1(xi))logμm(xi)

The tissue class images were automatically derived via the “new segmentation” algorithm in SPM8 (Ashburner and Friston, 2005). Default settings were used for the tissue segmentation, except that a non-parametric representation of the tissue intensity distributions was used, rather than the default mixture of Gaussians. The tissue class images used for estimating the deformations were at an isotropic resolution of 1.5 mm.

Following tissue classification, the diffeomorphic registration was repeated using two different regularisation settings.^1^ An elastic operator was used in both cases, as defined by:

(36) $$||Lv|{|}^{2}=\int_{x\in \Omega }\;\left(\frac{\lambda_{1}}{4}||Dv+{\left(Dv\right)}^{T}|{|}^{2}+\lambda_{2}||\text{tr}(Dv)|{|}^{2}+\lambda_{3}||v|{|}^{2}\right)dx$$

The three hyper-parameters control the following:

• *λ*_1_ penalises the amount of stretching and shearing (but not rotation).
• *λ*_2_ controls the divergence of the initial velocity, which in turn determines the amount of volumetric expansion and contraction.
• *λ*_3_ simply penalises absolute displacements. It is included to ensure the uniqueness of the resulting **K** operator.

The settings used were *λ*_1_ = 1.0, *λ*_2_ = 0.5, *λ*_3_ = 0.001 (referred to as GS1 in the results tables) and *λ*_1_ = 0.5, *λ*_2_ = 1.0, *λ*_3_ = 0.001 (called GS2).

A further set of registrations were also carried out, but using the one-parameter subgroup representation (Arsigny et al., 2006, 2009) of Dartel (Ashburner, 2007), rather than GS. The overall procedure was identical to GS2, except for the parameterisation of the deformations.

After registration, the results include a set of mappings from the template to each of the individuals. For the evaluation, mappings from each individual to each other individual were required, so that structure labels from each subject could be overlaid on images of all other subjects. These mappings were derived by composing the inverse of one mapping, with another mapping, and using the result to warp the structure labels from one subject into alignment with the anatomy of another.

The first of the datasets was from the Internet Brain Segmentation Repository (IBSR) provided by the Center for Morphometric Analysis at Massachusetts General Hospital.^2^ They consist of 18 anonymised T1-weighted MR scans (subject, scanner and sequence information are unknown), on which 43 individual structures have been manually labelled. The registration was based on the simultaneous alignment of grey matter, white matter, CSF, bone and soft tissue.

The second dataset is from the LONI Probabilistic Brain Atlas (LPBA40) (Shattuck et al., 2008)^3^ and consists of 40 skull-stripped T1-weighted images (with cerebellum and brain-stem removed), that have 56 structures manually delineated. Because the LPBA40 set had been closely skull-stripped, this registration was based only on simultaneous alignment of grey and white matter.

**Fig. 7:**
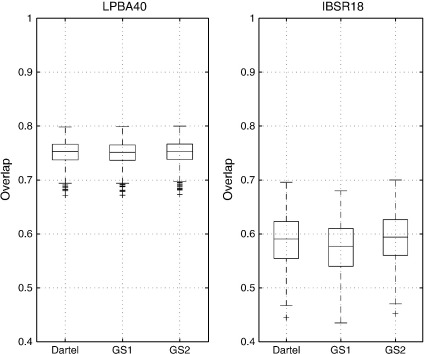
Volume overlap measures compare favourably with those obtained from the other registration algorithms evaluated in Klein et al. (2009) (this figure may be compared directly with Fig. 5 of that paper). On each box, the central mark is the median, the edges of the box are the 25th and 75th percentiles, the whiskers extend to the most extreme data-points not considered outliers. Any outliers are plotted individually.

The resulting target overlaps are shown in Fig. 7, and compare favourably with the best overlap results of Klein et al. (2009). For the IBSR18 dataset, the mean and median overlaps were 0.573 and 0.577 respectively for GS1, and 0.590 and 0.594 for GS2. Mean and median overlaps from the Dartel approach were 0.586 and 0.591. The greatest median overlap reported by Klein et al. (2009) was about 0.55, whereas the overlap from an affine registration (Jenkinson et al., 2002) was 0.40. For IBSR40, the mean and median overlaps were 0.750 and 0.751 for GS1, and 0.751 and 0.753 respectively for GS2. Mean and median overlaps from the Dartel approach were 0.751 and 0.753, very similar to the results from GS2. The highest median overlap reported by Klein et al. (2009) was 0.73, and that from affine registration was 0.60.

For these data, the overlaps obtained from the GS approach are not much greater than those obtained from Dartel. The principal reason for this is that the nonlinear displacements were all relatively small (less than about 8.5 voxels anywhere in any of the brains) because the data had first been affine registered together. Evaluations with larger displacements are presented later in the paper.

**Fig. 8:**
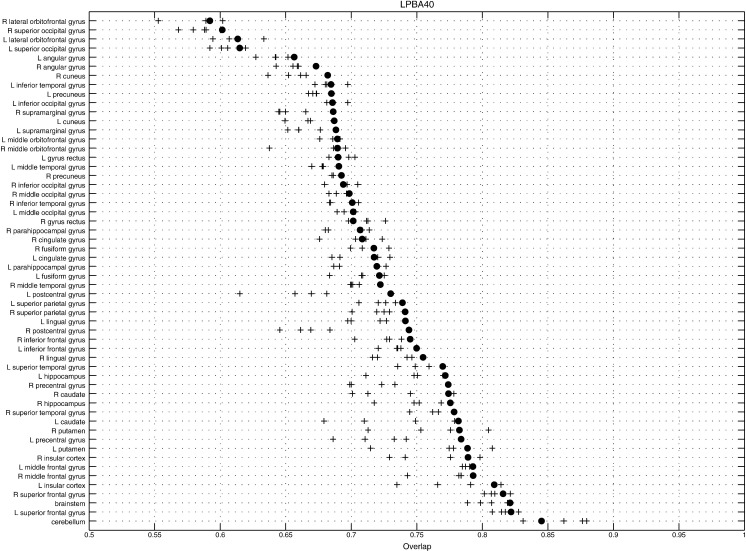
Average volume overlap for each region in the LPBA40 dataset (GS2). Results from the current GS approach are shown with filled circles. Crosses indicate results from the four algorithms evaluated in Klein et al. (2009) that performed best for this dataset (ART (Ardekani et al., 1995), SyN (Avants and Epstein, 2008), FNIRT (Andersson et al., 2007) and JRD-fluid (Chiang et al., 2007)).

**Fig. 9:**
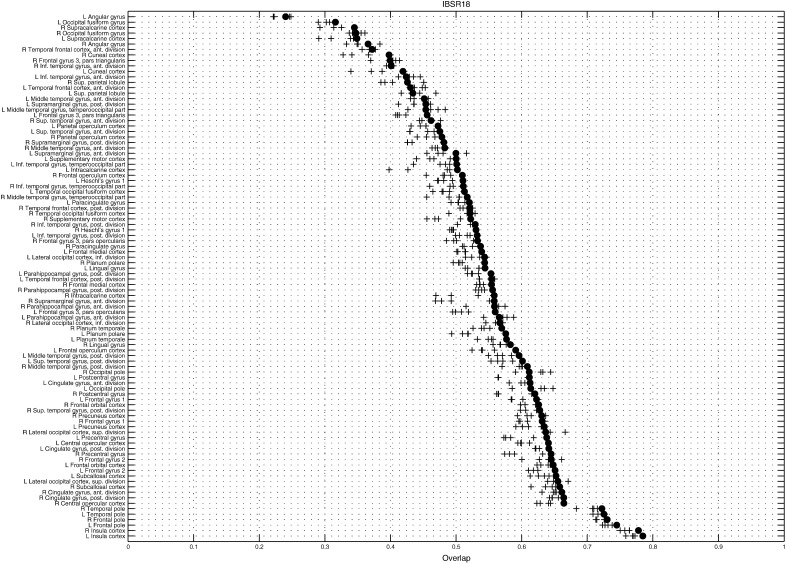
Average volume overlap for each region in the IBSR18 dataset (GS2). Results from the current GS approach are shown with filled circles. Crosses indicate results from the four algorithms evaluated in Klein et al. (2009) that performed best for this dataset (SPM_D (Ashburner, 2007), SyN (Avants and Epstein, 2008), IRTK (Rueckert et al., 2006) and ART (Ardekani et al., 1995)).

Using the affine registration as a baseline, the results showed 15% to 20% greater accuracy improvements^4^ when compared to those achieved for the most accurate of the nonlinear registration algorithms evaluated previously. These evaluations also showed that relatively small changes to the operator used to regularise the registration, can impact the final accuracies. Further exploration of the types of operators used, along with their various possible settings, could probably yield greater registration accuracy, but this was not the main aim of this work. Average overlaps (GS2) are shown for different brain structures^5^ in Figs. 8 and 9. Again, the plots show reasonably good overlap for the current method, compared to the best of the other algorithms.

There are some aspects of this evaluation, which some may claim do not provide a fair comparison against other methods. The first of these is that a group-wise registration scheme was used, and that this may have some “unfair” advantage over pairwise alignments. Certainly, there are advantages in terms of internal consistency among all the deformations, as well as execution times. However, as the main aim of inter-subject alignment is to align groups of subjects together, it would seem reasonable to try to achieve this using the most accurate strategy possible. Because the Dartel results were very similar to those from GS, the accuracy improvements demonstrated here seemed largely a result of the groupwise registration of tissue class images, rather than the way the deformations were parameterised.

**Fig. 10:**
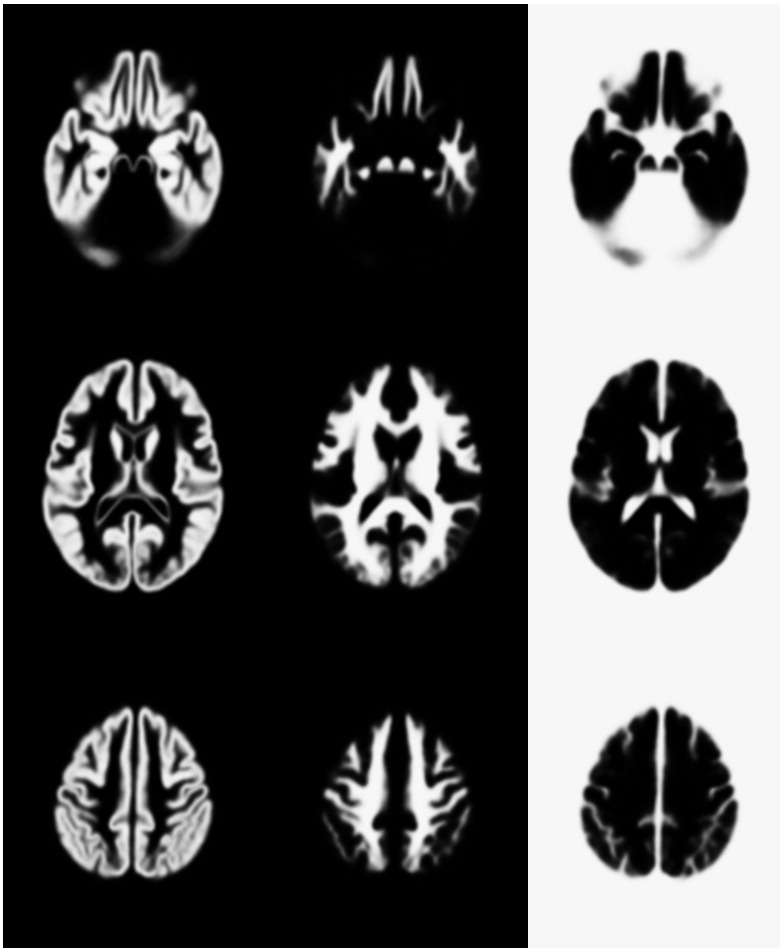
The LPBA40 tissue probability template, showing slices 40, 60 and 80 (GS2).

**Fig. 11:**
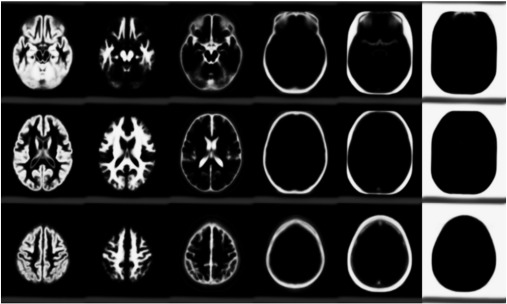
The IBSR tissue probability template, showing slices 40, 60 and 80 (GS2).

The second potential criticism may be that the evaluations were done by the authors, rather than an “impartial” investigator. Occasionally, evaluations by other parties may be more about the competence of the investigator to run the approach, rather than of the algorithms themselves. As the alignments were based on matching tissue classes together, the output from the initial segmentations were visually examined beforehand, as these have a strong influence on the final results. In practice though, the algorithms were not adjusted in order to increase the accuracy for these particular datasets, and everything was run without any manual adjustments of the data (such as manual re-orienting). Figs. 10 and 11 show the templates resulting from the two datasets after registration.

### Evaluation of convergence in 3D

One of the benefits of optimisation strategies that use second, as well as first, derivatives (such as Gauss–Newton or Levenberg–Marquardt) is that convergence is often much faster than approaches that use only the first derivatives (such as gradient descent). Here, convergence is assessed by plotting the value of the objective function with each iteration of the algorithm.

**Fig. 12:**
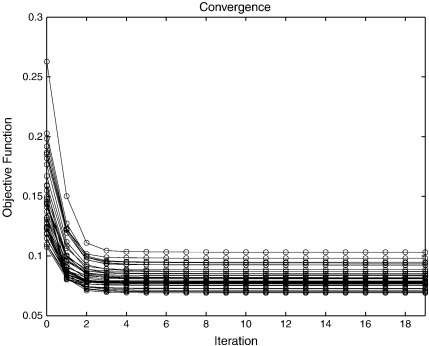
Objective function after different numbers of Gauss-Newton iterations, when matching the images in the LPBA40 dataset to their average.

In the previous subsection, a coarse-to-fine strategy was used, with the aim of avoiding some of the potential local minima. In this section, there is no coarse-to-fine strategy and eight time steps are used for the integration of the deformations. The template (see Fig. 10) and regularisation were fixed to that used for the final iterations in the evaluations in the previous section. The convergence for each of the subjects in the LPBA40 dataset was assessed, and plots of the objective function for each iteration are shown in Fig. 12.

**Fig. 13:**
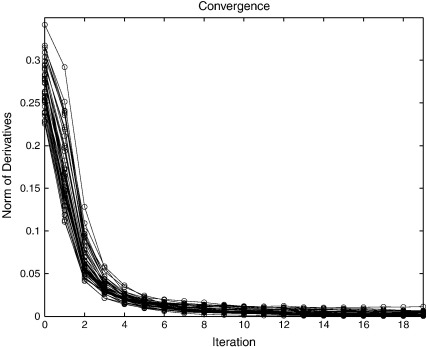
The norm of the derivatives of the objective function after different numbers of Gauss–Newton iterations. In principle, the norm should be zero if the algorithm has fully converged.

The *L*_2_ norm of the objective function gradients also provide a measure of convergence, so these are plotted in Fig. 13. In theory, these gradients should approach zero at the solution. This situation is not quite achieved in practice using the pure Gauss–Newton procedure (with *γ* fixed at 1 in Eq. (29)). The most likely reason for this is that the data are sampled discretely in the space of the template, leading to aliasing of high spatial frequency signal. This can cause the updates to overshoot slightly, causing the parameters to “bounce around” slightly for some regions of the images. Regularisation of the form described earlier (by reducing *γ* in Eq. (29)) could have been used to ensure that these norms properly approach zero. This is not shown, as the aim was to demonstrate the behaviour with a pure Gauss–Newton algorithm.

Gradient descent algorithms often require hundreds of iterations to achieve convergence. This component of the evaluation was intended to show that reasonable convergence may be achieved with about 10 iterations of a Gauss–Newton algorithm. Slightly more exact solutions may be achieved by decreasing the update steps, although more iterations may be required.

Each iteration of the GS approach is slower than for many other registration algorithms. On a Dell Precision T3500,^6^ each iteration took 43 s, whereas a Gauss–Newton iteration of Dartel (with six squaring steps) takes 20 s. The algorithm is of a type that should allow straightforward parallelisation, so further improvements could be achieved by implementing the most computationally intensive steps on GPUs.

### Evaluation with larger displacements

In the previous evaluations, all subjects’ brains were relatively healthy and of similar ages, so the impacts of much larger displacements were not really investigated. Those evaluations also involved images that had been first aligned together via 12-parameter affine transforms. For morphometric applications, the aim is usually to consider both shape and size, in which case the registration may be initialised using a rigid-body alignment. To assess the effects of larger displacements, the IBSR40 images were all translated along the anterior-posterior direction by 12 mm (8 voxels), and re-registered with the template previously generated from un-translated versions of the data (GS2). The length of an adult human brain varies with a standard deviation of about 8 mm, so 12 mm may be a typical displacement required for nonlinear registration of rigidly aligned brains. Velocity fields resulting from the translated scans were compared with those estimated from un-translated data, quantifying similarities between parameterisations $w_{a}$ and $w_{b}$ using correlation coefficients computed by

(37) $$r_{\mathrm{ab}}=\frac{w_{a}^{T}Aw_{b}}{\sqrt{w_{a}^{T}Aw_{a}}\sqrt{w_{b}^{T}Aw_{b}}}$$

where A is the large sparse matrix encoding the operator $L^{†}L$. To reduce the penalty against absolute displacements, the values of *λ*_1_, *λ*_2_ and *λ*_3_ used were 0.5, 1.0 and 0.00001 respectively. These are the same as for GS2, but with a much lower value for *λ*_3_. Registrations of displaced data were done twice: once with initial velocity estimates set uniformly to zero (to provide poor starting estimates), and once with them set uniformly to 8 voxels (providing good starting estimates). Because no coarse to fine strategy was used, the first experiment assesses the robustness of the alignments with respect to initial misregistration, whereas the second assesses the properties of the deformation model. For all cases, 20 Gauss–Newton iterations of the registration algorithm were used.

The mean correlation coefficient between results from the GS approach done without translations, versus the results of GS2 (from the previous evaluations), was 0.98. Ignoring the fact that the regularisation was slightly different in terms of penalising absolute displacements, this result showed that the coarse-to-fine strategy only played a small role in the comparison with human expert segmentation.

**Fig. 14:**
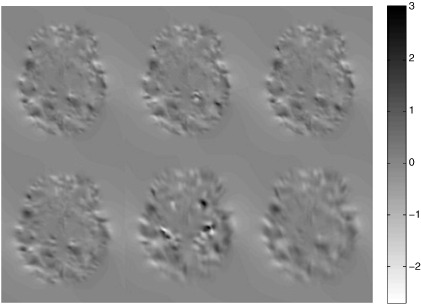
A single slice through the divergence of velocity fields computed after registering one of the LPBA40 subjects. The top row shows results from registering via GS, whereas the bottom row shows results from using Dartel. Results from un-translated data are shown (left column), followed by results of translated images with poor starting estimates (middle column) and finally results from translated data with close starting estimates (right column).

The mean correlation coefficient between results from the Dartel and GS approaches, using un-translated data was 0.84. This is a reasonably high correlation, which suggests that when displacements are small, results obtained by registering using Dartel or GS are reasonaby similar to each other. However, a comparison between registration results from un-translated and translated data tells a different story. Using uniformly zero starting estimates, the correlation coefficients for GS were 0.52, whereas those for Dartel were only 0.19. This showed a highly significant difference between the behaviour of the two approaches. No coarse-to-fine strategy was used for these registrations, so much of the difference is likely to result from getting cought in local optima. By repeating the registration with starting estimates that encode a uniform displacement of 12mm, many of these local minima were avoided. With closer starting estimates (of the sort that the coarse-to-fine approach may help provide), the correlation coefficients were increased to 0.98 and 0.47 for GS and Dartel respectively. These clearly indicate the superiority of formulating registration using the LDDMM or GS framework, rather than that of Dartel. A comparison between Fig. 2 and the left-side panel of 5 illustrates where the discrepancies arise, and Fig. 6 also illustrates the differing behaviours of the two models. Fig. 14 shows divergences (trace of Jacobian tensors) of the various estimated velocity parameterisations for one subject (S40). The slice has an axial orientation and contains the anterior cingulate. The thing to observe from the figure is that the GS results are all more similar to each other than those from Dartel, and that the Dartel results from aligning translated scans (lower centre and lower right) shows a clear blurring along the directionof translation. The fact that Dartel may be less ideally suited for computational anatomy studies was mentioned in Ashburner (2007).

## Conclusions

This work demonstrates that convergence of diffeomorphic registration can be speeded up with Gauss–Newton optimisation, and that the memory costs previously incurred by storing the entire sequence of velocity fields can be avoided. This overcomes some of the obstacles that currently hinder the widespread adoption of a more coherent computational anatomy framework. Although the alignment accuracy achieved from an implementation of this approach appears to be higher than that of other algorithms evaluated using the same datasets, further improvements in terms of the choice of differential operator etc should lead to even greater accuracy. The geodesic shooting algorithm is released as a toolbox for SPM8.^7^

## Footnotes

1 No other settings were tried.

2 Data available via http://www.cma.mgh.harvard.edu/ibsr/.

3 Data available via http://www.loni.ucla.edu/Atlases/Atlas_Detail.jsp?atlas_id=12.

4 (0.594 − 0.55)/(0.594 − 0.40) × 100*%* = 22.7*%* and (0.753 − 0.73)/(0.753 − 0.60) × 100*%* = 15.0*%*.

5 Overlap measures for other algorithms were obtained by dividing values in http://www.mindboggle.info/papers/evaluation_NeuroImage2009/tables/table_ROIxMethod_TO_LPBA40.csv and http://www.mindboggle.info/papers/evaluation_NeuroImage2009/tables/table_ROIxMethod_TO_IBSR18.csv by 100.

6 MATLAB (R2009a) benchmarks: LU 0.27; FFT 0.24; ODE 0.20; Sparse 0.80.

7 www.fil.ion.ucl.ac.uk/spm/software/spm8/.
